# The Effect of a Multicomponent Dual-Task Exercise on Cortical Thickness in Older Adults with Cognitive Decline: A Randomized Controlled Trial

**DOI:** 10.3390/jcm9051312

**Published:** 2020-05-02

**Authors:** Seongryu Bae, Kenji Harada, Sangyoon Lee, Kazuhiro Harada, Keitaro Makino, Ippei Chiba, Hyuntae Park, Hiroyuki Shimada

**Affiliations:** 1 Department of Preventive Gerontology, Center for Gerontology and Social Science, National Center for Geriatrics and Gerontology, Obu 474-8511, Japan; harada-k@ncgg.go.jp (K.H.); sylee@ncgg.go.jp (S.L.); harada@harbor.kobe-u.ac.jp (K.H.); kmakino@ncgg.go.jp (K.M.); ichiba@ncgg.go.jp (I.C.); htpark@dau.ac.kr (H.P.); shimada@ncgg.go.jp (H.S.); 2 Graduate School of Human Development and Environment, Kobe University, Rokkodai-cho, Nada-ku, Kobe 657-8501, Japan; 3 Department of Health Care Science, Dong-A University, Saha, Busan 604-714, Korea

**Keywords:** cortical thickness, dual-task, cognitive training, physical exercise, randomized controlled trial

## Abstract

The aim of this study was to examine cortical thickness changes associated with a multicomponent exercise intervention combining physical exercise and cognitive training in older adults with cognitive decline. This study involved a secondary analysis of neuroimaging data from a randomized controlled trial with 280 older adults having cognitive decline who were randomly assigned to either a multicomponent exercise group (*n* = 140) that attended weekly 90-minute exercise and cognitive training sessions or a health education control group (*n* = 140). The cortical thickness and cognitive performance were assessed at the baseline and at trial completion (10 months). The cortical thickness in the frontal and temporal regions was determined using FreeSurfer software. Cognitive performance was evaluated using the Gerontology-Functional Assessment Tool (NCGG-FAT). The cortical thickness significantly increased in the middle temporal (*p* < 0.001) and temporal pole (*p* < 0.001) in the multicomponent exercise group compared with the control group. Cortical thickness changes were significantly associated with change in trail making test (TMT)-A, TMT-B, and story memory after a 10-month multicomponent exercise intervention. This study suggests that multicomponent exercise programs combining physical exercise and cognitive training have important implications for brain health, especially in providing protection from age-related cortical thinning.

## 1. Introduction

According to the World Alzheimer Report, an estimated 46.8 million people worldwide had dementia in 2015, and this number is proposed to reach 131.5 million by 2050 [1]. Pharmacological treatment is relatively ineffective in halting the progression of dementia. Therefore, substantial research efforts are focusing on the use of non-pharmacological interventions for dementia, including cognitive training, physical exercise, and music therapy [2,3,4]. Non-pharmacological interventions may maintain or decrease the rate of cognitive decline in adults with mild cognitive impairment (MCI) and early stage dementia [5].

Physical and cognitive activities represent important modifiable protective factors for cognitive decline and dementia. Thus, physical and cognitive interventions have the potential to prevent cognitive decline [6]. The National Institutes of Health consensus panel on dementia reported that physical activity is the only intervention with sufficient evidence for recommendation as a preventative measure for cognitive decline [7]. Evidence suggests that physical exercise improves cognitive function [8,9] and increases hippocampal volume, and grey and white matter volume density in both healthy older adults and adults diagnosed with MCI [10,11,12]. Further, positive associations between cognitive activity, improved cognitive function, and lower risk of dementia have been reported [13,14]. Previous studies have reported that cognitive training improving cognitive function in older adults is related to the inhibition of hippocampal atrophy [15] and reduced brain beta-amyloid burden [16].

Recent meta-analytic reviews indicate that combined multimodal physical and cognitive training may result in greater improvement in cognitive function than either physical or cognitive interventions alone [17,18,19]. Further, a recent systematic review suggests that combined physical and cognitive intervention in older adults with MCI improves cognitive functions in multiple domains (e.g., global cognition, memory, executive function, and attention) more so than isolated physical exercise and cognitive groups [20]. Dual-task paradigms require the ability to simultaneously divide attention between two tasks, such as completing verbal fluency tasks while walking [21]. Older adults show declined dual-task performance due to age-related deterioration in cognitive function such as deficits in divided attention [14,22]. On the other hand, multicomponent exercise interventions including dual-task training seem to be beneficial for maintaining cognition and for reducing whole brain atrophy in older adults with amnestic MCI [23]. Although the present study did not comprise a multi-armed design, the strength of a multi-armed study design such as this one is the ability to distinguish the specific contribution of combined interventions from the effects of single physical exercise or cognitive groups [17]. The positive effects associated with combined physical exercise and cognitive training over single components could be attributed to the potential additive effects on neurogenesis, resulting from the initial pro-proliferative priming by the exercise component and the subsequent survival-promoting effects induced by cognitive challenges from the cognitive component [24,25,26]. However, it remains unclear whether multicomponent exercise interventions under dual-task physical exercise and cognitive training are effective at inducing changes in the cortical surface such as cortical thickness. 

Cortical thickness is calculated as the mean distance from the grey–white matter boundary to the grey–cerebrospinal fluid boundary within a defined cortical region [27]. It is thought to indicate the genesis of neurons within cortical columns to better reflect cortical features such as the size and density of neuronal cell bodies, organization of cortical layers, and synaptic connections [28,29]. Many studies have confirmed extensive age-related differences in cortical thickness [30], suggesting an age-dependent cortical volumetric reduction. Two studies examined the effects of aerobic exercise intervention on cortical thickness, but their results were in disagreement [31,32]. Jiang and colleagues have reported that cognitive training affects changes in cortical thickness in the healthy elderly [33].

Evidence suggests that exercise may moderate age-related brain atrophy in a regionally selective manner [34]. Colcombe et al. (2006) found that aerobic fitness training involving a 6-month walking intervention in sedentary older adults led to significant increases in brain volume in the frontal and temporal regions [10]. Reiter et al. (2015) reported that improvements in cardiovascular fitness (CRF) with a 12-week aerobic exercise regimen were associated with increased cortical thickness in the superior temporal gyrus in older adults with MCI [32]. Randomized controlled trials (RCT) have shown that cognitive training can increase cortical thickness in the frontal region, including the lateral orbitofrontal cortex and frontal pole regions [33,35]. Furthermore, age-associated volume reductions have been reported to be more pronounced in the frontal lobe compared to other brain regions [36,37], while there are also studies that have found that the frontal and temporal lobes age at similar rates [38]. Based on these findings, in this study, we focused on the frontal and temporal regions. We predicted that these would undergo changes from exercise or cognitive training and would be reduced in older adults with cognitive impairment due to the progression of cognitive decline and cortical thinning [39].

To the best of our knowledge, there is yet to be a thorough investigation into the effectiveness of multicomponent exercise combining physical exercise and cognitive training (i.e., requiring cognitive loads during physical exercise) on cortical thickness in older adults with cognitive decline. The current study aimed to examine cortical thickness changes associated with a 10-month single-blinded RCT of multicomponent exercise intervention under dual-task conditions in older adults with cognitive decline. We hypothesized that multicomponent exercise intervention combining physical exercise and cognitive training would increase cortical thickness of the frontal and temporal regions over the 10-month trial period. Furthermore, we hypothesized that changes in cortical thickness would be positively associated with changes in cognitive performance.

## 2. Materials and Methods

### 2.1. Participants and Procedures

**Figure 1:**
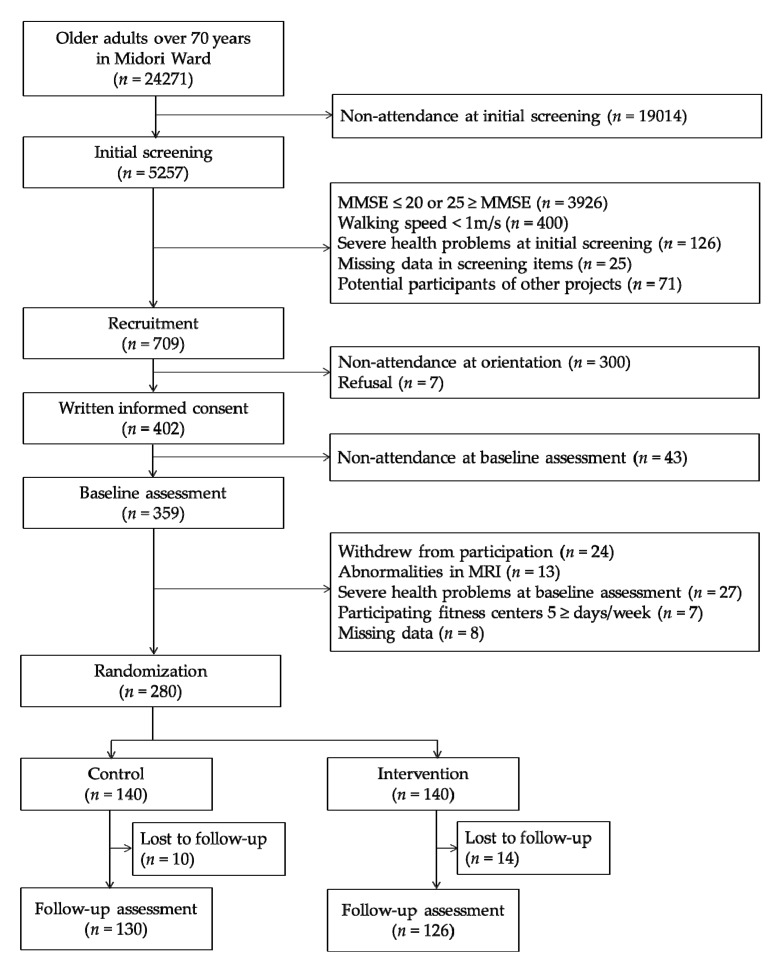
Flowchart of the participants’ recruitment process. MMSE, Mini-Mental State Examination; MRI, Magnetic resonance imaging.

The present study was a secondary analysis of a 10-month single-blinded RCT of multicomponent exercise for older adults with global cognitive decline. The protocol for this trial (ID: UMIN000013097) was registered in the University Hospital Medical Information Network Clinical Trials Registry website (http://www.umin.ac.jp/ctr/index.htm). The main results of this trial are being prepared for submission as another manuscript. Several papers on the secondary analysis of this trial have already been published elsewhere [40,41]. The participants of the trial were recruited from a subcohort of the National Center for Geriatrics and Gerontology Study of Geriatric Syndromes (NCGG-SGS) [42], conducted in 2013 in Midori Ward of Nagoya city, Aichi Prefecture, Japan. A total of 24,271 individuals were invited to participate in the screening survey of physical and cognitive function; 5257 of these individuals participated. Of the 5257 participants, 709 were selected as potential participants after applying the following inclusion criteria: (1) scores between 21 and 24 at screening on the Mini-Mental State Examination (MMSE) reflecting global cognitive decline [43]; (2) did not have severe health problems including dementia, stroke, depression, and Parkinson’s disease; (3) normal gait speed ≥1 m/s; and (4) were not enrolled in other intervention studies. Recruitment documents were sent to the 709 individuals selected, and 359 participated in the baseline assessment. Additionally, after baseline assessment, 79 individuals were excluded who (1) withdrew from participation; (2) had abnormalities in magnetic resonance imaging (MRI); (3) were newly diagnosed with dementia, stroke, depression, or Parkinson’s disease; (4) attended fitness centers for more than 5 days per week; and (5) had missing data. Finally, 280 participants were assigned to either the intervention or control group using a computerized randomization scheme with a 1:1 ratio by a researcher blind to the study aims (Figure 1).

In the intervention group, multicomponent exercise programs were provided in 90-minute sessions once a week for 40 weeks. Participants in the health education control group attended three health education classes (90 min each) while sitting during the 10-month study period. These classes, conducted by a professional lecturer, consisted of information on the prevention of dependence on long-term care, oral care, and strategies for healthy longevity. The classes did not contain any specific information regarding physical exercise or cognitive activities. After the 40-week program, follow-up assessments were conducted for both groups.

The trial complied with the Declaration of Helsinki and was approved by the Ethics Committee of the National Center for Geriatrics and Gerontology (Approval Number: 637-3). Written informed consent was obtained from all participants.

### 2.2. Intervention

The multicomponent exercise program consisted of weekly 90-minute classes of aerobic exercise, physical exercise and cognitive combined dual-task training, muscle strength training, and balance training for the 10-month intervention period. The program took place in three fitness facilities located in the area under the instruction of a professional fitness instructor. Before intervention, we discussed the program content with the instructors to create this multicomponent exercise program. Each 90-minute session for 19–32 participants consisted of a 15-min warm-up, muscle strength training for 20 min, aerobic and cognitive dual-task training for 20 min, aerobic exercise for 20 min, and ending with a 15-min balance training and cool-down period. Before each exercise session, participants underwent a health check and attendance record. Aerobic exercises included stair stepping, endurance and brisk walking, and aerobic dance. The heart rate was recorded after aerobic exercise in each session to measure pulse. With reference to previous studies, the mean exercise intensity was set to approximately 60% of the maximum heart rate [8,44]. For dual-task training, participants simultaneously performed cognitive tasks while exercising. These included walking or stair-stepping while counting numbers forward or backward in order, starting from one; walking or stair-stepping while calculating numbers; walking or stair-stepping while clapping on multiples of three; walking or stair-stepping while executing a word chain (i.e., saying a word that started with the last letter of the mentioned word); walking or stair-stepping while reciting a poem; walking or stair-stepping while talking; and walking while reciting a sentence backward. In order to avoid familiarization with the cognitive tasks, participants were challenged with new and increasingly difficult cognitive tasks as the session progressed. In addition, to promote exercise and behavioral changes, participants performed home-based multicomponent exercise programs, practicing and self-monitoring using worksheets and accelerometers.

### 2.3. Neuropsychological Test Battery

Objective cognitive functions were assessed using the National Center for Geriatrics and Gerontology-Functional Assessment Tool (NCGG-FAT) [45]. These tasks involved story memory tests by immediate and delayed recognition. Participants listened to a short story and were instructed to remember its details. Then, they had to immediately answer 10 questions pertaining to the story (immediate recognition). The questions were repeated after 20 min and had to be answered correctly (delayed recognition). We calculated the composite score using the sum of delayed recognition and immediate recognition (range: 0 to 20). Attention and executive function were assessed by the Trail Making Test Part A and B (TMT-A and TMT-B) [46]. For TMT-A, participants were asked to touch the target numbers displayed randomly on the monitor in consecutive order (1–15). For TMT-B, participants were asked to touch target numbers and letters (Japanese Kana characters) as quickly as possible. We recorded the time (in seconds) taken to complete each test. The processing speed was assessed by symbol-digit substitution test (SDST) in which nine pairs of numbers and symbols were displayed at the top of the monitor [47]. A target symbol was present in the center of the monitor. Participants were asked to choose the number corresponding to the target symbol at the bottom of the monitor. The score was the number of correct answers chosen within 120 s.

Depressive symptoms were measured using the 15-item Geriatric Depression Scale (GDS-15) [48].

### 2.4. MRI Acquisition

Structural magnetic resonance imaging (MRI) was acquired on a Siemens MAGNETOM Trio Tim 3T scanner (Siemens Medical Solutions, Erlangen, Germany) with 12 channel head coils. A whole brain three-dimensional T1-weighted magnetization prepared rapid acquisition gradient echo (MPRAGE) sequence was acquired in the sagittal plane: repetition time (TR) = 1800 ms, echo time (TE) = 1.99 ms, flip angle = 9˚, slices = 160, slice thickness = 1.1 mm, voxel = 1.0 × 1.0 × 1.1 mm, image matrix = 256 × 256 mm, and field of view (FOV) = 250 mm. Each scan took four minutes six seconds.

### 2.5. Regional Cortical Thickness Measurement

Surface-based morphometry was performed using the neuroimaging package FreeSurfer version 6.0 (http://surfer.nmr.mgh.harvard.edu/) on the Ubuntu operating system version 16.4 Long Term Support (LTS). This image processing suite provided automated segmentation of cortical and subcortical brain structures and calculated cortical thickness. Fischl (2012) documented details of the image processing procedure [49]. Briefly, all images were automatically processed using skull stripping, spatial transformation, atlas registration, and spherical surface maps. After tessellating the grey–white boundary and locating the grey–pial boundary in FreeSurfer, cortical thickness was calculated as the closest distance between the grey–white matter boundary and the pial mesh at each vertex on the tessellated surface [27]. After automatic segmentation, scans were visually checked; no changes were made following the manual checks. Automated cortical segmentation and region of interest (ROI) definition were performed using the Desikan–Killiany Atlas, which manually labelled 34 cortical ROIs in each hemisphere [50]. Based on our hypotheses, we focused on cortical thickness in ROIs in the frontal lobe (superior frontal, rostral middle frontal, caudal middle frontal, lateral orbitofrontal, medial orbitofrontal, frontal pole, and precentral) and temporal lobe (superior temporal, middle temporal, inferior temporal, fusiform, temporal pole, and parahippocampal).

### 2.6. Aerobic Fitness

In order to investigate the association between changes in cortical thickness and aerobic fitness, we assessed aerobic fitness capacity using the 6-minute walk test (6MWT). Participants were required to walk as fast as possible in 6 min along a straight 10-m path; the total distance (in meters) walked in 6 min was assessed. A longer distance indicated a higher aerobic fitness level. The 6MWT has sufficient reliability and validity and is safe and easily measured to obtain an estimate of aerobic fitness in older adults [51].

### 2.7. Statistical Analysis

Baseline characteristics between the intervention and control groups were compared using the t-test and Chi-square test. The analyses were performed to evaluate the between- and within-group effects on the cortical thickness of each ROI in the 10-month intervention period using a repeated-measures mixed model design. Independent categorical variables (fixed factors) were groups (control and intervention groups), measurement time (baseline and 10-month follow-up), and the interaction between groups and measurement time. When the repeated-measures mixed models indicated that measurement time and group interaction was significant, tests of simple main effects using a Bonferroni correction were performed to determine which group or groups differed significantly across the intervention period. The effect size was calculated using Cohen’s d. The effect sizes were considered large when d = 0.8 or higher, were considered intermediate when d = 0.50–0.70, and were considered small when d = 0.20–0.40 [52]. In the regions where group by time interaction effects were significant, Pearson’s correlation analysis was performed for the changes in cortical thickness and changes in cognitive performance and aerobic fitness within each group. In addition, linear regression analysis was performed to assess the relationship between changes in cortical thickness and changes in cognitive performance and aerobic fitness while controlling for age, gender, years of education, geriatric depression scale (GDS), and estimated total intracranial volume (eTIV) to minimize confounding influences, and standardized β values were calculated. To analyze changes over time, we computed cognitive performance delta scores (10-month follow-up minus baseline), 6MWT performance delta scores (10-month follow-up minus baseline), and cortical thickness delta scores (10-month follow-up minus baseline). All analyses were conducted using IBM SPSS Statistics software package (25.0; SPSS Inc., Chicago, IL, USA). The level of statistical significance was set at *p* < 0.05.

## 3. Results

### 3.1. Baseline Characteristics and Completion Rates

Baseline characteristics of the study participants are shown in Table 1. In total, 280 participants were scanned at baseline; 23 dropped out or were excluded for poor imaging quality. A total of 256 participants were included in the secondary analysis, with 130 control participants and 126 in the intervention group. There were no significant differences between groups in terms of age, gender, years of education, GDS score, eTIV, or cognitive performance. Participant attendance rates of 83% and 77% were recorded for the intervention and control groups, respectively.

### 3.2. The Effects of Training on Cortical Thickness

Table 2 summarizes the mean difference between baseline and postintervention values, the effect within time and between groups, and the interaction of time and groups on cortical thickness between the two groups. Results indicated a significant group by time interaction effect in the left middle temporal (F (1, 268) = 17.082; *p* < 0.001) and left temporal pole region (F (1, 256) = 16.364; *p* < 0.001). Cortical thickness of the left middle temporal (*p* < 0.001) and left temporal pole (*p* < 0.001) were significantly increased compared to baseline in the intervention group but not in the control group. There were no group by time interaction effects on cortical thickness in other regions.

### 3.3. Association Between Changes in Cortical Thickness and Cognitive Performance

**Figure 2:**
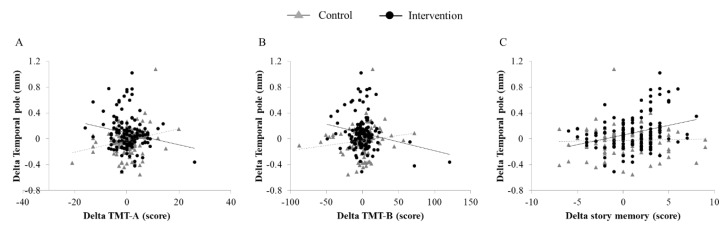
Relationship between changes in cortical thickness of the left temporal pole and cognitive performance: The change in thickness in the left temporal pole is plotted against change in cognitive performance (**A**) TMT-A, (**B**) TMT-B, and (**C**) story memory. Observations from the control group are triangles, and the regression line is dashed. Observation from the intervention group are circles, and the regression line is linear. TMT: trail making test.

Correlation analyses were run separately for the control and intervention groups (Table 3). In the intervention group, the change in TMT-A (*r* = −0.182; *p* = 0.042), TMT-B (*r* = −0.194; *p* = 0.030), and story memory (*r* = 0.313; *p* = 0.001) were significantly associated with increased cortical thickness in the left temporal pole (Figure 2). In addition, the change in story memory was significantly associated with increased cortical thickness in the left middle temporal region (*r* = 0.236; *p* = 0.008). These associations remained significant after adjusting for age, sex, education, and eTIV, except for the association between SDST and the left temporal pole. In the control group, the change in TMT-A was positively associated with changes in cortical thickness of the left temporal pole (*r* = 0.235; *p* = 0.008).

### 3.4. Association Between Changes in Cortical Thickness and Aerobic Fitness

Pearson’s correlation and linear regression analyses revealed no significant correlations between the 6MWT and left middle temporal region (*r* = −0.008, *p* = 0.464; *β* = 0.046, *p* = 0.597) and left temporal pole (*r* = 0.094, *p* = 0.150; *β* = 0.138, *p* = 0.120) in the intervention group. Similarly, there were no significant correlations between the 6MWT and left middle temporal region (*r* = −0.054, *p* = 0.277; *β* = −0.085, *p* = 0.363) and left temporal pole (*r* = 0.039, *p* = 0.334; *β* = 0.045, *p* = 0.626) in the control group.

## 4. Discussion

The present study revealed that, in community-dwelling older adults with cognitive decline, 10-month multicomponent exercise under dual-task conditions significantly increased cortical thickness of the left temporal lobe (i.e., middle temporal and temporal pole) relative to that of the control group. Furthermore, changes in cortical thickness were significantly associated with changes in cognitive performance after receiving multicomponent exercise interventions for 10 months. Specifically, changes in cortical thickness of the left middle temporal and left temporal poles were positively associated with changes in story memory performance. These regions are involved in several cognitive processes, including language processes, semantic memory processing, and integrating spatial information from different sensory signals. Moreover, reduced cortical thickness is associated with worsening performance on a wide variety of neuropsychological tests [53,54]. A previous study reported that slower TMT-B completion time is associated with decreased cortical thickness in the frontal and temporal regions and in the inferior parietal lobe, suggesting that TMT performance is involved in the temporal lobe [55]. However, in this study, the relationship between TMT-A and cortical thickness in the temporal pole showed inconsistent results in the intervention and control groups. In the intervention group, the change in TMT-A was significantly associated with increased cortical thickness in the left temporal pole. On the other hand, in the control group, the change in TMT-A was associated with decreased cortical thickness in the left temporal pole. It is difficult to interpret these inconsistent results. A possible reason for the inconsistency in our study is that TMT-A performance might not be associated with changes in cortical thickness in the temporal pole that decrease with age. Previous studies have reported that TMT-B, which requires the participants to draw lines to connect circled numbers and letters in an alternating numeric and alphabetic sequence, is more difficult compared to TMT-A [56]. Because TMT-A is relatively simple, it is possible that the performance was likely to change. On the other hand, it is possible that the control group did not depend on cortical thickness in the temporal pole. Compared with healthy controls, individuals with Alzheimer’s disease (AD) or MCI demonstrate more cortical thinning in the temporal lobe [57,58]. We observed stronger associations between multicomponent exercise under dual-task condition and changes in left temporal lobe cortical thickness in older adults with cognitive decline, suggesting that intervention-induced neuroplasticity in this region may prove the multicomponent exercise-mediated cognitive performance benefits. 

To the best of our knowledge, this is the first study to examine the association between exercise, cortical thickness, and cognitive function in older adults with cognitive decline after multicomponent exercise intervention under dual-task condition. Brain atrophy is a predictor of cognitive impairment [59], and research has focused on cortical thinning as an important predictor of cognitive impairment [53,54]. Cortical thickness is an effective biomarker for tracking cognitive and neuropathological symptoms of dementia [60,61], including amyloid beta protein and tau deposition [62], as well as future conversion to AD [63]. The most prominent regions of brain atrophy in older adults with MCI include the hippocampus, entorhinal cortex [64], inferior and medial temporal cortices, precuneus, posterior cingulate, and temporoparietal junction [65]. Although cortical thinning is recognized as a marker of AD progression, there is scant evidence that non-pharmacological interventions may slow the rate of brain atrophy progression in these regions in cognitively impaired older adults. 

Cross-sectional studies have suggested that older adults with higher levels of cardiovascular fitness (CRF) have greater cortical thickness than their lower CRF peers [66] and that more sedentary non-demented individuals have less medial temporal lobe thickness [67]. Only a few studies have examined the effects of aerobic exercise intervention on cortical thickness, and results have been equivocal. Improvements in CRF with a 12-week walking intervention were associated with increased cortical thickness in the left insula and superior temporal gyrus in older adults with MCI [32]. However, this study examined a small sample size (14 MCI and 16 healthy controls) in a non-randomized controlled trial. Another 6-month RCT study of aerobic exercise in sedentary healthy older adults did not detect cortical thickness differences between groups but reported changes in cognitive performance and cortical thickness in the dorsolateral prefrontal cortex. It was concluded that changes in cortical thickness induced by aerobic exercise are slow and may be undetectable within 6 months [31]. Our study employed a relatively long-term intervention period compared to those in previous studies, thereby enabling detection of changes in cortical thickness. 

The multicomponent exercise program in the current study examined not only aerobic exercise but also the effects of a dual-task program combining physical exercise and cognitive training. Our previous multicomponent exercise trial demonstrated reduced whole brain atrophy and improved logical memory test performance in older adults with amnestic MCI, highlighting the potential cognitive and neural benefits of a multicomponent exercise program [23,44]. Previous studies proposed that physical exercise, particularly aerobic exercise, has positive effects on brain structure and cognitive functions through several underlying mechanisms. First, physical exercise regulates a number of growth factors including brain-derived neurotrophic factor (BDNF), which plays a crucial role in neuroprotection and synaptic plasticity [68,69], and insulin-like growth factor 1 (IGF−1), which promotes neuronal growth and improves cognitive performance [70,71]. Second, physical exercise educes inflammatory cytokines and oxidative stress, which suggest anti-inflammatory and antioxidant effects on the brain [72,73]. Third, aerobic exercise can promote cerebral blood flow, which enhances neurogenesis and improves cognition [74,75]. Although cognitive training has been related to better cognitive performance and increasing brain volumes in older adults, neural mechanisms underlying the protective effects of these cognitive activities are largely unknown. One possible explanation for better cognitive performance by cognitive training might be a use-dependent plasticity of synaptic strength and brain structure [76,77]. Older adults with higher engagement in cognitive activities can provide stimulation for the cognitive system. Furthermore, according to the hypothesis of cognitive reserve, the enriched environment, through cognitive activities, can influence a neural processing and synaptic organization by allowing neurological processes to become more efficient and adaptive [78]. Both physical exercise and cognitive training are considered to affect cognitive function as well as brain structure and function via shared underlying mechanisms for neural plasticity (i.e., synaptogenesis, angiogenesis, and neurogenesis) [79]. The simultaneous dual-task physical exercise and cognitive training may build upon the strength of each intervention while concomitantly offsetting its relevant weakness to maximize the beneficial effects of these interventions on brain structure and function due to their synergistic neural effects [25,80,81]. In other words, multicomponent dual-task exercise that requires a cognitive load during physical exercise may produce greater neural and cognitive benefits than exercise alone. Although there are limited studies combining physical exercise and cognitive training, these studies reported that the combined intervention produced larger improvements in working memory than did physical exercise or cognitive training alone [82,83]. Our results support these previous findings and clarify that cortical thickness is increased in older adults with cognitive decline through a 40-week multicomponent exercise intervention including cognitive loads during physical exercise. 

This study has several strengths. The large sample size, multicomponent intervention design and long-term follow-up period constitute clear strength, as does the assessment of the intervention-induced enhancement in cortical thickness and cognitive performance in community-dwelling older population. Additionally, the randomized trial design helped to minimize biases from known and unknown potential confounders and to draw causal inferences. However, this study has several limitations that should be kept in mind when interpreting the results. First, we did not find associations between cortical thickness and aerobic fitness (i.e., 6MWT). One possible explanation for this may be the method of aerobic fitness measurement. In this study, aerobic fitness was measured by the 6MWT and did not include physiological measures of aerobic fitness such as maximal oxygen uptake (i.e., VO_2_ max). However, considering our participants, we had to adopt a safer measurement. Second, despite the apolipoprotein E (APOE) ε4 being a genetic risk factor for neurodegenerative diseases such as AD and cognitive decline [84], we did not measure genetic risk factors. However, baseline characteristics were not significantly different between groups in cognitive performance. The third limitation is related to the FreeSurfer’s processing pipeline. In order to reduce within-subject noise, an unbiased, robust, within-subject template was created between the two time points of each participant. A longitudinal processing pipeline was proposed in FreeSurfer, but we did not use it. Hence, it is possible that within-subject noise occurred. Another limitation is the possibility of including participants with normal cognition among the participants of this study. In this study, participants with MMSE scores between 21 and 24 at screening were defined as having global cognitive decline. Although most studies used a cutoff score of 23/24 or below to indicate cognitive impairment [43,85,86], cutoff was not consistent across studies. Other studies proposed that scores of 0–9 correspond to severe cognitive impairment, 10–20 to moderate impairment, 21–24 to mild impairment, and 25–30 to questionable impairment or intact function [87,88]. A recent systematic review reported that pooled estimates across 15 studies (*n* = 12,796) resulted in a sensitivity of 0.89 (95% CI, 0.85 to 0.92) and a specificity of 0.89 (95% CI, 0.85 to 0.93) of the MMSE to detect dementia at a cutoff point of 23/24 or less [89]. Lastly, it is possible that the improvement seen in the intervention group resulted from the social interaction that the intervention group received in the study period. This possibility cannot be completely excluded with the present design and should be addressed in future studies.

## 5. Conclusions

In conclusion, a 10-month multicomponent exercise program combining aerobic exercise and cognitive training is an effective intervention to increase cortical thickness of the temporal lobe in older adults with cognitive decline. Furthermore, changes in cortical thickness were associated with changes in cognitive performance after multicomponent exercise interventions, suggesting that multicomponent exercise-induced neuroplasticity in the temporal lobe may mediate beneficial effects on cognitive function. Further studies are needed to determine whether the observed effects are maintained and to elucidate the mechanisms underpinning changes in cortical thickness induced by multicomponent dual-task exercise.

## Figures and Tables

**Table 1:** Baseline characteristics of study participants.

	Control (*n* = 140)	Intervention (*n* = 140)	*p*-Value
Age (years)	76.4 ± 4.2	76.3 ± 4.1	0.761
Gender (female), %	37.1	42.1	0.392
Year of education (years)	12.1 ± 2.5	11.7 ± 2.6	0.150
GDS (score)	2.6 ± 2.3	2.8 ± 2.2	0.441
eTIV (cm^3^)	1399.6	1385.5	0.421
6MWT (m)	450 ± 53	453 ± 60	0.610
Cognitive performance			
TMT-A (sec)	20.9 ± 4.9	20.9 ± 5.2	0.943
TMT-B (sec)	43.2 ± 21.9	42.4 ± 17.0	0.740
SDST (score)	51.6 ± 9.7	52.6± 10.3	0.382
Story memory composite (score)	11.9 ± 3.4	11.8 ± 3.7	0.724

Values indicate mean ± standard deviation (SD) or number (%). All *p*-values were generated from a t-test or chi-square test. GDS, geriatric depression scale; eTIV, estimated total intracranial volume; 6MWT, 6-minute walk test; TMT, trail making test; SDST, symbol-digit substitution test.

**Table 2:** Cortical thickness per group and time.

		Mean Difference (95% CI) Between Baseline and Postintervention Values		Group	Time	Group × Time	Cohen’s d
	Hemi	Intervention (*n* = 126)	Control (*n* = 130)	*p* ^a^	*p* ^a^	*p* ^a^	
Frontal lobe, mm							
Superior frontal	L	−0.008 (−0.017 to 0.001)	−0.007 (−0.016 to 0.001)	0.904	0.017	0.928	0.011
	R	−0.008 (−0.017 to 0.001)	−0.005 (−0.013 to 0.003)	0.551	0.027	0.519	0.081
Rostral middle frontal	L	−0.009 (−0.018 to 0.001)	−0.003 (−0.011 to 0.006)	0.898	0.063	0.308	0.128
	R	0.004 (−0.005 to 0.014)	0.004 (−0.005 to 0.013)	0.965	0.195	0.971	0.004
Caudal middle frontal	L	−0.007 (−0.019 to 0.005)	−0.007 (−0.019 to 0.005)	0.267	0.102	0.991	0.004
	R	−0.004 (−0.017 to 0.009)	−0.007 (−0.020 to 0.005)	0.087	0.209	0.724	0.044
Lateral orbitofrontal	L	−0.006 (−0.019 to 0.007)	0.003 (−0.009 to 0.016)	0.672	0.795	0.312	0.127
	R	−0.011 (−0.028 to 0.006)	0.009 (−0.008 to 0.026)	0.731	0.858	0.106	0.204
Medial orbitofrontal	L	−0.009 (−0.023 to 0.005)	0.009 (−0.006 to 0.023)	0.137	0.98	0.09	0.214
	R	0.000 (−0.015 to 0.016)	0.012 (−0.003 to 0.027)	0.693	0.254	0.292	0.132
Frontal pole	L	0.006 (−0.023 to 0.036)	0.004 (−0.025 to 0.033)	0.488	0.609	0.915	0.013
	R	0.013 (−0.015 to 0.041)	0.035 (0.007 to 0.063)	0.826	0.017	0.269	0.139
Precentral	L	0.001 (−0.012 to 0.014)	−0.002 (−0.015 to 0.011)	0.859	0.913	0.706	0.047
	R	−0.007 (−0.024 to 0.009)	−0.003 (−0.020 to 0.014)	0.4	0.382	0.713	0.046
Temporal lobe, mm							
Superior temporal	L	0.002 (−0.013 to 0.018)	−0.017 (−0.032 to −0.002)	0.954	0.185	0.08	0.221
	R	−0.010 (−0.019 to 0.000)	−0.013 (−0.023 to −0.004)	0.856	0.001	0.634	0.06
Middle temporal	L	0.095 (0.060 to 0.130)	−0.008 (−0.043 to 0.026)	0.002	0.001	<0.001	0.519
	R	−0.006 (−0.016 to 0.005)	−0.010 (−0.020 to 0.001)	0.902	0.039	0.594	0.067
Inferior temporal	L	−0.007 (−0.029 to 0.005)	−0.009 (−0.021 to 0.003)	0.134	0.071	0.839	0.025
	R	−0.003 (−0.016 to 0.010)	0.002 (−0.011 to 0.015)	0.095	0.886	0.63	0.06
Fusiform	L	−0.003 (−0.018 to 0.011)	−0.002 (−0.016 to 0.012)	0.679	0.594	0.902	0.015
	R	−0.007 (−0.020 to 0.006)	0.005 (−0.008 to 0.017)	0.15	0.783	0.203	0.16
Temporal pole	L	0.086 (0.045 to 0.127)	−0.032 (−0.073 to 0.008)	0.104	0.069	<0.001	0.508
	R	−0.035 (−0.070 to 0.000)	−0.045 (−0.079 to −0.011)	0.702	0.001	0.69	0.05
Parahippocampal	L	−0.030 (−0.050 to −0.011)	−0.032 (−0.051 to −0.013)	0.063	0	0.901	0.016
	R	−0.006 (−0.023 to 0.011)	−0.007 (−0.024 to 0.010)	0.901	0.312	0.944	0.009

^a^ Repeated measure mixed model analysis. Hemi, hemisphere; L, left; R, right.

**Table 3:** Relationship between change in cortical thickness and cognitive performance in control and intervention groups.

	Control Group								Intervention Group							
	Δ Left Middle Temporal				Δ Left Temporal Pole				Δ Left Middle Temporal				Δ Left Temporal Pole			
	r	*p* ^a^	β	*p* ^b^	r	*p* ^a^	β	*p* ^b^	r	*p* ^a^	β	*p* ^b^	r	*p* ^a^	β	*p* ^b^
Δ TMT-A	−0.014	0.872	0.002	0.984	0.235	0.008	0.223	0.013	−0.084	0.351	−0.082	0.347	−0.182	0.042	−0.179	0.041
Δ TMT-B	0.005	0.959	−0.007	0.937	0.143	0.11	0.123	0.178	−0.060	0.503	−0.123	0.164	−0.194	0.03	−0.266	0.003
Δ SDST	0.09	0.314	0.101	0.272	0.06	0.506	0.033	0.721	0.164	0.068	0.152	0.086	0.182	0.042	0.142	0.116
Δ Story memory	0.09	0.317	0.111	0.229	0.035	0.695	0.025	0.785	0.236	0.008	0.212	0.017	0.313	0.001	0.277	0.002

^a^ Pearson’s correlation analysis. ^b^ Linear regression analysis controlling for age, gender, years of education, geriatric depression scale, and estimated total intracranial volume. TMT: trail making test; SDST: symbol-digit substitution test; Δ, delta score.
